# Marathon Run-induced Changes in the Erythropoietin-Erythroferrone-Hepcidin Axis are Iron Dependent

**DOI:** 10.3390/ijerph17082781

**Published:** 2020-04-17

**Authors:** Maja Tomczyk, Jakub Kortas, Damian Flis, Barbara Kaczorowska-Hac, Agata Grzybkowska, Andzelika Borkowska, Ewa Lewicka, Alicja Dabrowska-Kugacka, Jędrzej Antosiewicz

**Affiliations:** 1 Department of Bioenergetics and Nutrition, Faculty of Physical Education, Gdansk University of Physical Education and Sport, 80-336 Gdansk, Poland; maja.tomczyk@awf.gda.pl (M.T.); agata.p3@gmail.com (A.G.); 2 Department of Recreation and Qualify Tourism, Faculty of Tourism and Recreation, Gdansk University of Physical Education and Sport, 80-336 Gdansk, Poland; jakub.kortas@awf.gda.pl; 3 Department of Bioenergetics and Nutrition, Faculty of Rehabilitation and Kinesiology, Gdansk University of Physical Education and Sport, 80-336 Gdansk, Poland; damian.flis@awf.gda.pl; 4 Departament of Occupational Therapy, Faculty of Rehabilitation and Kinesiology, Gdansk University of Physical Education and Sport, 80-336 Gdansk, Poland; barbara.kaczorowska@awf.gda.pl; 5 Department of Bioenergetics and Physiology of Exercise, Medical University of Gdansk, 80-210 Gdansk, Poland; andzelika.b@gumed.edu.pl; 6 Department of Cardiology and Electrotherapy, Medical University of Gdansk, 80-210 Gdansk, Poland; elew@gumed.edu.pl (E.L.); alidab@gumed.edu.pl (A.D.-K.)

**Keywords:** ferritin, body iron stores, *HFE* mutation, HMGB1

## Abstract

Alterations in iron metabolism after physical activity are manifested through the rise of blood hepcidin (Hpc) levels. However, in many athletes, no changes in Hpc levels are observed after exercise despite the presence of inflammation. The missing links could be erythropoietin (EPO) and erythroferrone (ERFE), which down-regulate Hpc biosynthesis. EPO, ERFE and Hpc biosynthesis is modified by serum iron through transferrin receptor 2. Consequently, we investigated whether marathon-induced changes in EPO, ERFE and Hpc levels are blood iron-dependent. Twenty-nine healthy male marathon runners were analyzed. Serum iron, ferritin, transferrin, EPO, ERFE and Hpc levels were assessed before, immediately after, and 9 ± 2 days after the marathon. The runners whose serum Hpc decreased after the marathon (*n = 15*), showed a significant increase in ERFE levels. In athletes whose serum iron levels were below 105 µg/day (*n = 15*), serum EPO (*p = 0.00*) and ERFE levels (*p = 0.00*) increased with no changes in Hpc concentration. However, in athletes with low serum iron, no changes in EPO levels were observed when serum ferritin exceeded 70 ng/mL (*n = 7*). Conversely, an increase in ERFE levels was observed in marathoners with low serum iron, independently of serum ferritin (*n = 7*). This indicates modulation of blood iron may affect exercise-induced changes in the EPO/ERFE/Hpc axis. Further study is needed to fully understand the physiological meaning of the interdependence between iron and the EPO/ERFE/Hpc axis.

## 1. Introduction

Physical activity has been shown to alter systemic and cellular iron metabolism. The main effect of exercise on iron metabolism seems to be mediated by hepcidin (Hpc), a hormone which inhibits duodenal iron absorption through its release from intracellular stores to the blood. Exercise-induced increases in Hpc concentrations have been reported [1,2]. In addition, according to some studies, many individuals experience excess body iron accumulation, which could be partially associated with lack of exercise [3]. Iron accumulation in many organs, including the liver, heart, skeletal muscles and the endocrine system, may lead to a series of health disorders [4,5,6]. Conversely, high levels of exercise are associated with low body iron stores [3]. Furthermore, regular training decreases body iron stores both in professional athletes and recreationally active elderly people [7,8,9,10]. The exact mechanism of how exercise reduces body iron stores remains unknown. One of the possible explanations is that exercise increases blood hepcidin levels, diminishing the intestinal iron absorption [11]. Furthermore, a possible factor that could predispose iron accumulation is hereditary hemochromatosis [12]. It is one of the most common human genetic disorders, estimated to affect 1 in 300 people in populations of Northern European origin [13]. The three most common mutations responsible for the development of hemochromatosis are found in the *HFE* gene located on Chromosome 6. Since the HFE protein mediates intracellular signaling pathways which result in increased Hpc biosynthesis, it is of interest to determine whether athletes who harbor mutations in the *HFE* gene would respond the same way to exercise as runners harboring the wild-type (WT) *HFE* gene. Moreover, another possible explanation for the exercise-induced reduction of the body iron stores is the increase in erythropoiesis. In this case, stored iron (in ferritin) is incorporated into hemoglobin. The process of erythropoiesis is controlled by a number of factors, such as erythropoietin (EPO) and erythroferrone (ERFE), a newly discovered hormone [14]. Their metabolic effects are strictly related to iron metabolism and EPO has been shown to stimulate ERFE, which, in turn, reduces Hpc biosynthesis [14]. On the cell level, EPO gene expression is under the control of hypoxia inducible factor 1 alpha (HIF1α). Low oxygen tension or decreased kidney interstitial fibroblast iron can lead to HIF1α activation and increase EPO synthesis. In addition, an interconnection between hormones of EPO/ERFE/Hpc and iron metabolism is underlined by action of transferrin receptor 2 (Tfr2) which appears to function as a sensor of circulating iron. Tfr2 regulates Epo sensitivity and, in an iron-replete state, it restricts Epo action [15]. Furthermore, hepatic TfR2 promotes iron signaling to stimulate hepcidin synthesis and inhibit iron fluxes to thebloodstream [16]. These data clearly indicate that both blood iron and intracellular iron may influence the effects of exercise on the EPO/ERFE/Hpc signaling axis.

The purpose of the current study was to reexamine the outcomes of a marathon run on the serum EPO-ERFE-Hpc signaling axis. In addition, the effects of *HFE* mutation, inflammation, serum iron concentration and iron stores on this axis were evaluated.

## 2. Materials and Methods

### 2.1. Experimental Approach to the Problem

To assess the marathon-induced changes in iron metabolism, the blood was sampled three times: 1 month before the marathon after a 3-d rest (Pre samples); immediately after the marathon run (Post1 samples); and 39 9 ± 2 days after the competition (Post2 samples).

Fasting blood was collected at the Pre and Post2 time points, in the morning; at the Post1 time point, the blood was collected immediately after the runners reached the finish line. Blood samples for hormones measurement were collected from the antecubital vein into a disposable vacutainer. Immediately after collection, the samples were centrifuged at 2000× *g* at 4 °C, for 10 min. The serum was transferred to new tubes and stored at −80 °C prior to biochemical analysis.

### 2.2. Subjects

41 amateur marathon runners took part in the study, however, 29 participants completed all stages of the experiment (mean age 40 ± 8 years).

Avarage marathon time obtained by participants was 3 h, 44 min, and 35 s.

Subject’s characteristic and weekly training volume is presented in Table 1.

The study was officially approved by the Bioethical Committee of the Regional Medical Society in Gdansk (KB-24/16) and performed in compliance with the Declaration of Helsinki. Before commencing the marathon run and testing, the study was verbally described to the subjects. All runners provided a written informed consent to participate in the study. In addition, prior the study, the runners obtained a signed agreement from their physicians confirming the ability to participate in the marathon.

### 2.3. Biochemical Analysis

The blood was taken three times, at the Pre, Post1, and Post2 time points. Total iron-binding capacity (TIBC),the iron, ferritin and leukocytes levels were measured at Synevo Medical Laboratory (Gdansk, Poland). The serum Hpc, ERFE, and high-mobility group box 1 protein (HMGB1) levels were determined using the appropriate Cloud-Clone Corp kits (Katy, TX, USA).

Quantitative determinations of EPO in the serum were performed automatically using IMMULITE 2000 EPO, a solid-phase enzyme-labeled chemiluminescent immunometric assay (IMMULTITE 2000, Siemens). Potential changes in the hematocrit value were taken into consideration in data presentation.

### 2.4. Analysis of the Hemochromatosis HFE Gene

For the analysis, human DNA was isolated using the High Pure Polymerase Chain Reaction (PCR) Template Preparation Kit (Roche) (Belmont, CA, USA). The *HFE* genotype was determined using LightMix Kit HFE H63D S65C C282Y (TibMolbiol) and LightCyclerFastStart DNA Master HybProbe (Roche) (Belmont, CA, USA). The former is an in vitro diagnostic test, which enables the detection and identification of clinically-relevant single-nucleotide polymorphism variants p.H63D, p.S65C, and p.C282Y of the *HFE* gene. Two fragments of the *HFE* gene were PCR-amplified simultaneously using specific oligonucleotide fragments. Fluorescently labeled probes were used to identify the PCR products.

The genotype was determined by performing a melting curve analysis. The 284-bp PCR fragment containing the c.845G>A (C282Y) polymorphism was analyzed using a LightCycler Red 640 fluorophore. The 163 bp PCR fragment containing the c.197C>G (H63D) or c.193A>T (S65C) polymorphism was analyzed using a Simple Probe 519 fluorophore. Real-time PCR was performed using Light Cycler 2.0 (Roche) (Belmont, CA, USA).

### 2.5. Statistical Analyses

Statistical analysis was performed using Statistica 12.0 software (Statsoft, Tulsa, OK). All values are expressed as the mean ± standard deviation (*SD*). A Shapiro–Wilk test was used to assess the homogeneity of dispersion from the normal distribution. A Brown–Forsythe test was used to evaluate the homogeneity of variance. For homogenous results, an analysis of variance (ANOVA) for repeated measures and post-hoc Tukey’s test were performed to identify significantly different results. For heterogeneous results, ANOVA Friedman’s test and the right post-hoc test were applied. The significance level was set at *p < 0.05*.

## 3. Results

The mean levels of iron, ferritin and transferrin were within normal range in all athletes, except two who presented decreased serum iron and transferrin levels (Table 2). Analysis of the *HFE* gene revealed that 11 of 29 runners were heterozygotes for H63D mutation; 18 runners harbored the WT *HFE* gene. No differences were noted in some study parameters (iron, ferritin, Hpc and ERFE levels) of the runners harboring WT and H63D *HFE* gene before and after the marathon (data not shown). However, one week after the marathon run, a slight increase in ERFE levels was observed in the WT*HFE* gene group (0.17 ± 0.12 vs. 0.24 ± 0.15, *p = 0.01*). The serum levels of Hpc, the main regulator of iron metabolism, were measured before and after the marathon. Interestingly, seven of the H63D mutation carriers demonstrated an increase in Hpc levels after the marathon, while four showed a decrease. Furthermore, an increase in the markers of inflammatory response and muscle damage (creatine kinase (CK), leukocytes, neutrophils, and pentraxin 3) was apparent. However, contrary to the expectations, the levels of HMGB1, a pro-inflammatory protein [17], did not increase after the marathon. Finally, transferrin, TIBC and EPO levels increased after the marathon run (Table 2), which may indicate a stress-adaptive response induced by intense running (23,24).

No statistically significant changes in Hpc levels were apparent at the Post1 and Post2 time points. However, detailed analysis revealed that Hpc levels increased in 14 runners (responders) and decreased in the remaining runners. A subsequent detailed analysis of several runner subgroups was performed to understand the triggering factors of the observed changes. The runners were divided into two groups: the Hpc-level increase group (*n = 14*) and the Hpc-level decrease group (*n = 15*). Detailed analysis of these groups revealed significant changes in the Hpc Post 1 levels (Table 3). Furthermore, after the marathon run, the ERFE levels were significantly elevated in runners whose Hpc levels decreased (Table 3). The levels returned to the baseline values in Post2 measurements. At the completion of the race, an increase in transferrin levels was observed in both groups. Furthermore, the Post2 serum iron levels decreased in the Hpc-level increase group (Table 3).

**Figure 1:**
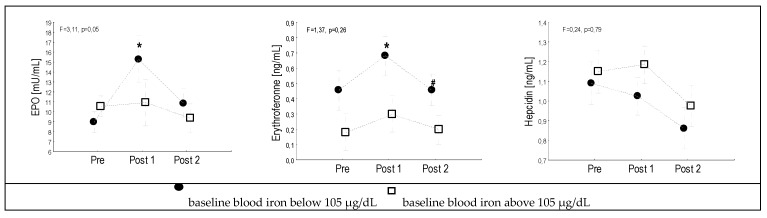
Blood iron levels determine the effect of marathon running on hormones involved in the iron metabolism. Legend: Pre—before the run, Post1—at the finish line, Post 2—9 ± 2 days after the run; * significantly different from the Pre measurement, # significantly different from the Post 1 measurement.

To evaluate the effect of the serum iron levels on marathon-induced changes in EPO, ERFE and Hpc levels, the runners were divided into two groups, based on serum iron concentrations, at which significant changes in the EPO, ERFE and Hpc parameters were observed. In the first group (*n = 15*), the serum iron pre-concentration was < 105 µg/dL (81 ± 21 µg/dL), while in the second group (*n = 14*), it was >105 µg/dL (146 ± 43 µg/dL). Interestingly, a significant increase in serum EPO and ERFE levels, and a slight reduction in Hpc levels were observed in the runners whose serum iron pre levels were below 105 µg/dL (Figure 1).

To assess the relationship between body iron stores (represented by serum ferritin), and EPO, ERFE and Hpc, the runners were divided into two groups: those with ferritin levels < 70 ng/mL (39 ± 21 ng/mL, *n = 13*) and those with levels > 70 ng/mL (122 ± 35 ng/mL, *n = 16*). EPO levels increased in the former group (Pre: 10.75 ± 4.59 mU/mL; Post1: 17.28 ± 11.61 mU/mL) but remained unchanged in the latter group (Pre: 8.7 ± 2.34; Post1: 10.4 ± 3.38). Furthermore, the EPO levels did not change in the runners with ferritin levels exceeding 70 ng/mL (*n = 7*), even when they were accompanied by serum iron levels below 105 µg/dL. Furthermore, a significant negative correlation was apparent between the baseline ferritin and EPO levels at the three time points analyzed (Pre: *r = −0.48*, *p = 0.03*; Post1: *r = 0.53*; *p = 0.01*; Post2: *r = 0.50*, *p = 0.02*). The serum iron levels were also negatively correlated with EPO levels after the marathon run (Post1: *r = 0.50*, *p = 0.02*; Post2, *r = 0.40*, *p = 0.05*). Conversely, ERFE levels increased only in athletes with serum iron levels below 105 µg/dL (*n = 15*), and the ferritin status did not affect the response to the marathon run (data not shown).

## 4. Discussion

In the present study, we demonstrated that alterations in the levels of hormones regulating the iron metabolism (EPO, ERFE, and Hpc) induced by a marathon run significantly depend on the serum iron and ferritin concentrations at the baseline. However, changes in the iron metabolism of athletes who are *HFE* H63D heterozygotes were similar to those in athletes who harbor the WT *HFE* gene. Hence, the initial hypothesis that Hpc levels in the former are different from the latter was not confirmed. Interestingly, most of the obtained results indicated that exercise increases serum Hpc levels. However, Hpc levels do not change after exercise in some athletes [18,19,20]. The underlying mechanism of this phenomenon remains unresolved. Since *HFE* mutations are common among the Caucasian population [13], we hypothesized that the Hpc response to exercise might be impaired in athletes who are *HFE* heterozygotes. In the current study, increased Hpc levels after the marathon run were noted for 14 runners, while Hpc levels decreased in 15 runners. *HFE* heterozygotes were identified in both groups. Hence, the data did not support the initial hypothesis.

In the current study, the only difference between the WT *HFE* gene and H63D gene carriers was a decrease in Hpc levels in the former one week after the marathon. It has been previously reported that the iron stores in young boys and girls who do not engage in a regular physical activity and harbor the H63D mutation are higher than in WT controls [21]. In the current study, we did not observe a statistically significant difference in the serum ferritin levels among groups. Previously, it has been shown that regular exercise results in a reduction of body iron stores (12,13), which could explain observations reported herein. It was also suggested that low serum ferritin levels are associated with the absence of Hpc response to exercise. In athletes, whose ferritin levels were below 30 ng/mL, no changes in Hpc concentration after exercise are noted [19]. In the current study, only four participants had ferritin levels below 30 ng/mL. An increase of Hpc levels upon exercise was noted in two of them. In addition, no correlation was apparent between serum ferritin and Hpc levels before and after the marathon run. Hpc is a hormone that inhibits iron absorption from the digestive tract and iron release from the intracellular stores [22]. Stimulated erythropoiesis results in increased iron demand which leads to Hpc down-regulation. The mechanism of Hpc down-regulation has been clarified recently by the discovery of a new hormone, ERFE, which inhibits hepatic Hpc biosynthesis [14]. Although it is generally known that regular exercise stimulates erythropoiesis, and the erythrocyte mass of the athletes is higher than in inactive individuals, to the best of our knowledge, no data explaining the effect of exercise on the blood ERFE levels in humans have been published. Data presented in the current study demonstrate that ERFE levels increase after the marathon run and remain elevated nine days later, but only in the runners whose Hpc levels decreased after the marathon run. This may suggest an inhibitory effect of ERFE on hepcidin biosynthesis [14]. In addition, the findings of the current study indicate that a reduction or, possibly, no changes in the Hpc levels after a race could be explained by increased ERFE levels. On the other hand, an increase in Hpc levels after the race cannot be explained by changes in ERFE levels, but, rather, by increased levels of pro-inflammatory cytokines [23]. Considering that extreme exercises, such as marathon running, stimulates pro-inflammatory cytokines production, it is possible that the iron metabolism is affected in some cells exposed to high cytokine levels [24,25]. The observed increase in pentraxin 3 and the number of leukocytes, confirmed that the marathon induced significant inflammation. Furthermore, detailed analysis revealed that athletes with low serum iron levels (<105 µg/dL) presented a significant increase in ERFE levels after the marathon, while ERFE levels were stable in those with high serum iron levels (>105 µg/dL). Interestingly, serum ferritin concentration, considered to be a good marker of the body iron stores [26], had no effect on the marathon-induced changes in ERFE levels.

**Figure 2:**
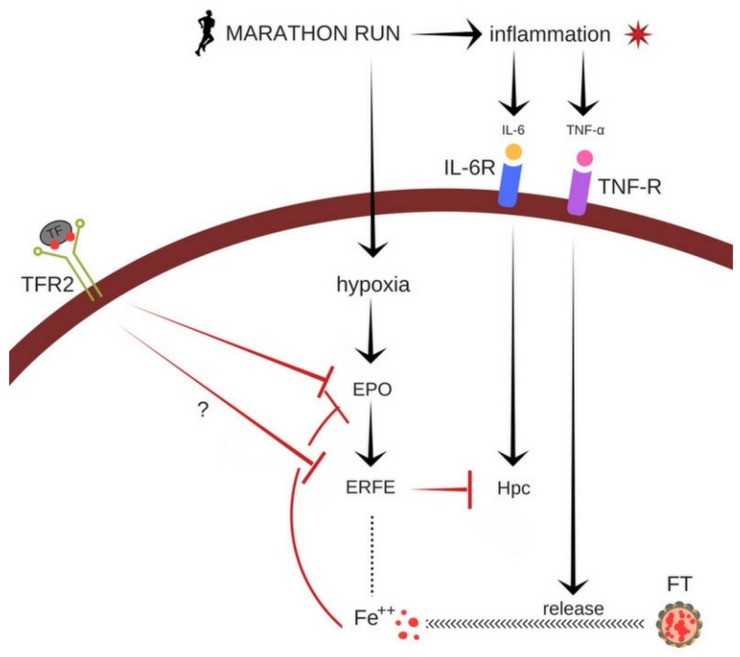
Summary of the potential effects of a marathon running on cellular iron metabolism.

EPO stimulates ERFE biosynthesis [14]. Hence, we anticipated that the iron status could influence ERFE synthesis via its effects on EPO concentration. The reported effect of a marathon run on serum EPO levels is unclear: it either increases, decreases or does not change after the run [27,28]. The mechanism of this phenomenon is also unknown. Data presented in the current study clearly indicate that the marathon-induced increase in EPO levels is determined by the serum iron levels and body iron stores. EPO levels increased in the runners with a low baseline concentration of serum iron and ferritin. In animal models, serum iron in the form of diferric transferrin triggers signaling through a transferrin receptor 2 (Tfr2). Tfr2 is expressed in the liver and erythroid cells. Blocking this receptor leads to increased EPO and ERFE production, and decreased Hpc biosynthesis [29]. It can be expected that, during a marathon run, the hypoxic signaling is augmented in runners with low serum iron levels and reduced in athletes with high serum iron levels. Indeed, increased EPO and ERFE levels were observed only in the runners with low blood iron levels. This is supported by the observation that exposure of iron-deficient patients to hypoxia increases serum EPO levels to a greater extent than in individuals with normal iron status [30]. Conversely, hypoxia-induced changes in Hpc levels are minute in iron-deficient patients [30]. In addition, iron can influence Hpc synthesis by triggering signaling which inhibits EPO synthesis. The summary of the potential effects of a marathon run on cellular iron metabolism is presented in Figure 2.

The figure shows the complexity of cellular iron metabolism. Extreme exercise, such as marathon running, induces inflammation, which can be measured, e.g., by the increase of the interleukin 6 (IL-6) levels in the blood which may lead to an increase in Hpc biosynthesis in the cell. By contrast, marathon run-induced hypoxia stimulates EPO and ERFE biosynthesis, which can inhibit of Hpc biosynthesis. Diferric transferrin binding to Tfr2, triggers signaling that leads to the inhibition of EPO and ERFE synthesis. This signaling is abrogated in athletes with relatively low blood iron levels. Exercise-induced inflammation can trigger ferritin degradation and iron release, which can inhibit EPO biosynthesis.

***→ - stimulation; –| - suppression; ..... - unknown effect; ? - possible effect***

## Figures and Tables

**Table 1:** General characteristics of participants.

	Baseline (*n = 29*)
**Age [years]**	39.29 ± 8.58
**Height [m]**	1.79 ± 0.05
**Body mass [kg]**	79.76 ± 7.50
**BMI [kg/m^2^]**	24.89 ± 2.13
**Training [h/week]**	6.39 ± 2.28
**Training [km/week]**	55.84 ± 19.77

Values are means ± SD; BMI–body mass index.

**Table 2:** The effects of marathon running on the inflammation and iron metabolism in young man.

	Pre	Post1	Post2
	(*n = 29*)	(*n = 29*)	(*n = 29*)
Fe [μg/dL]	113.57 ± 47.27	115.10 ± 31.67	114.34 ± 47.12
Ferritin [ng/mL]	84.79 ± 51.36	93.32 ± 61.90	80.22 ± 55.65
Transferrin [mg/dL]	277.21 ± 33.66	303.59 ± 39.75 ^a, *p = 0.01*^	278.34 ± 29.16 ^b, *p = 0.02*^
TIBC [μg/dL]	316.76 ± 41.92	345.38 ± 42.65 ^a, *p = 0.03*^	302.36 ± 41.03 ^b, *p = 0.00*^
ERFE [ng/mL]	0.27 ± 0.43	0.42 ± 0.43	0.30 ± 0.35
EPO [mU/mL]	9.60 ± 3.61	13.09 ± 8.37 ^a, *p = 0.03*^	10.27 ± 4.99
Hpc [ng/mL]	1.12 ± 0.39	1.09 ± 0.36	0.92 ± 0.38
HMGB-1 [ng/mL]	21.92 ± 3.91	21.17 ± 5.63	19.74 ± 4.06 ^a, *p = 0.04*^
Pentraxin3 [pg/mL]	250.28 ± 85.93	403.68 ± 258.45 ^a, *p = 0.00*^	166.70 ± 70.49 ^b, *p = 0.00*^
Leukocytes [G/L]	6.03 ± 1.14	16.82 ± 3.59 ^a, *p = 0.00*^	6.05 ± 1.88 ^b, *p = 0.00*^
Neutrophils [G/L]	3.27 ± 0.85	14.12 ± 3.18 ^a, *p = 0.00*^	3.35 ± 1.57 ^b, *p = 0.00*^
CK [U/L]	158.00 ± 76.26	411.14 ± 173.50 ^a, *p = 0.00*^	225.81 ± 142.49 ^b, *p = 0.00*^

Values are means (± SD); Fe-serum iron; TIBC-total iron binding capacity; ERFE-erythroferrone; EPO-erythropoietin; Hpc-hepcidin; HMGB-1-high-mobility group box 1 protein; CK-creatine kinase. Measurements: Pre–before the run; Post 1—at the finish line; Post 2—after 9 ± 2 days after the run; ^a^ significant differences compared to the Pre measurement; ^b^ significant differences compared to the Post 1 measurement.

**Table 3:** Marathon run-induced changes in serum hepcidin (Hpc) levels are related to the increase in serum ERFE levels.

	Group	Pre	Post 1	Post 2
Fe [μg/dL]	∆Hpc↑ (*n = 14*)	113.64 ± 54.60	115.64 ± 30.58	98.29 ± 39.78
	∆Hpc↓ (*n = 15*)	113.50 ± 40.73	114.60 ± 33.71	129.33 ± 49.73
Ferritin [ng/mL]	∆Hpc↑ (*n = 14*)	80.51 ± 46.16	88.84 ± 60.31	76.87 ± 59.26
	∆Hpc↓ (*n = 15*)	88.79 ± 57.11	97.49 ± 65.17	83.35 ± 53.96
Transferrin [mg/dL]	∆Hpc↑ (*n = 14*)	280.50 ± 40.72	310.21 ± 46.60 ^a, *p = 0.00*^	279.07 ±28.49 ^b, *p = 0.00*^
	∆Hpc↓ (*n = 15*)	274.13 ± 26.56	297.40 ± 32.53 ^a, *p = 0.00*^	277.67 ± 30.74 ^b, *p = 0.00*^
TIBC [μg/dL]	∆Hpc↑ (*n = 14*)	314.93 ± 47.53	352.07 ± 47.42 ^a, *p = 0.00*^	304.00 ± 35.91 ^b, *p = 0.00*^
	∆Hpc↓ (*n = 15*)	318.47 ± 37.56	339.13 ± 38.26	300.83 ± 46.53 ^b, *p = 0.00*^
ERFE [ng/mL]	∆Hpc↑ (*n = 14*)	0.40 ± 0.60	0.45 ± 0.57	0.36 ± 0.48
	∆Hpc↓ (*n = 15*)	0.16 ± 0.13	0.40 ± 0.27 ^a, *p = 0.00*^	0.24 ± 0.12 ^b, *p = 0.00*^
EPO [mU/mL]	∆Hpc↑ (*n = 14*)	9.34 ± 2.85	14.24 ± 9.65	8.91 ± 2.86 ^b, *p = 0.00*^
	∆Hpc↓ (*n = 15*)	9.85 ± 4.29	12.17 ± 7.42	11.53 ± 6.21
Hpc [ng/mL]	∆Hpc↑ (*n = 14*)	0.81 ± 0.23 †	1.09 ± 0.37 ^a, *p = 0.00*^	0.77 ± 0.28 ^b, *p = 0.00*^
	∆Hpc↓ (*n = 15*)	1.41 ± 0.28	1.09 ± 0.35 ^a, *p = 0.00*^	1.06 ± 0.42 ^a, *p = 0.00*^
HMGB-1 [ng/mL]	∆Hpc↑ (*n = 14*)	21.57 ± 3.51	20.67 ± 4.35	18.88 ± 4.35
	∆Hpc↓ (*n = 15*)	22.24 ± 4.34	21.64 ± 6.73	20.55 ± 3.74
Pentraxin3 [pq/mL]	∆Hpc↑ (*n = 14*)	266.24 ± 97.66	351.49 ± 238.99	171.72 ± 67.56
	∆Hpc↓ (*n = 15*)	235.46 ± 73.96	452.1 ±274.99 ^a, *p = 0.00*^	162.41 ± 75.17 ^b, *p = 0.00*^
Leukocytes [G/L]	∆Hpc↑ (*n = 14*)	5.86 ± 1.15	16.42 ± 3.57 ^a, *p = 0.00*^	6.10 ± 2.30 ^b, *p = 0.00*^
	∆Hpc↓ (*n = 15*)	6.21 ± 1.15	17.22 ± 3.70 ^a, *p = 0.00*^	6.00 ± 1.43 ^b, *p = 0.00*^
Neutrophils [G/L]	∆Hpc↑ (*n = 14*)	3.14 ± 0.73	13.75 ± 3.02 ^a, *p = 0.00*^	3.47 ± 2.00 ^b, *p = 0.00*^
	∆Hpc↓ (*n = 15*)	3.41 ± 0.96	14.48 ± 3.40 ^a, *p = 0.00*^	3.22 ± 1.04 ^b, *p = 0.00*^
CK [U/L]	∆Hpc↑ (*n = 14*)	144.92 ± 63.51	445.86±193.25 ^a, *p = 0.00*^	206.77 ±121.52 ^b, *p = 0.00*^
	∆Hpc↓ (*n = 15*)	165.79 ± 87.34	376.43±150.26 ^a, *p = 0.00*^	243.50 ±162.10 ^b, *p = 0.00*^

Values are means (± *SD*); Fe-serum iron; TIBC-total iron binding capacity; ERFE-erythroferrone; EPO-erythropoietin; Hpc-hepcidin; HMGB1-high-mobility group box 1 protein; CK-creatine kinase. Measurements Pre-before the run; Post1—at the finish line; Post2—9 ± 2 days after the run; ^a^ significantly different from the Pre measurement; ^b^ significantly different from the Post1 measurement; † significant differences between groups.

## Abbreviations

Hpc: hepcidin; ERFE: erythroferrone; EPO: erythropoietin; TrR2: transferrin receptor 2; *HFE*: homeostatic iron regulator; high Fe; WT: wild type; TIBC: total iron body capacity; HMGB1: high mobility group box 1; PCR: polymerase chain reaction; CK: creatine kinase; Fe: serum iron level; IL-6: interleukin 6.

## References

[1] Antosiewicz J., Kaczor J.J., Kasprowicz K., Laskowski R., Kujach S., Luszczyk M., Radziminski L., Ziemann E. (2013). Repeated “all out” interval exercise causes an increase in serum hepcidin concentration in both trained and untrained men. Cell. Immunol..

[2] Antosiewicz J., Ziolkowski W., Kaczor J.J., Herman-Antosiewicz A. (2007). Tumor necrosis factor-alpha-induced reactive oxygen species formation is mediated by JNK1-dependent ferritin degradation and elevation of labile iron pool. Free. Radic. Boil. Med..

[3] Balaban E.P., Snell P., Stray-Gundersen J., Frenkel E.P. (1995). The effect of running on serum and red cell ferritin. A longitudinal comparison. Int. J. Sports Med..

[4] Borkowska A., Sielicka-Dudzin A., Herman-Antosiewicz A., Halon M., Wozniak M., Antosiewicz J. (2011). P66Shc mediated ferritin degradation--a novel mechanism of ROS formation. Free. Radic. Boil. Med..

[5] Candau R., Busso T., Lacour J.R. (1992). Effects of training on iron status in cross-country skiers. Graefe’s Arch. Clin. Exp. Ophthalmol..

[6] Frise M.C., Cheng H.-Y., Nickol A.H., Curtis M.K., Pollard K.A., Roberts D.J., Ratcliffe P.J., Dorrington K.L., Robbins P.A. (2016). Clinical iron deficiency disturbs normal human responses to hypoxia. J. Clin. Investig..

[7] Ganz T., Nemeth E. (2006). Iron imports. IV. Hepcidin and regulation of body iron metabolism. Am. J. Physiol. Liver Physiol..

[8] Halon-Golabek M., Borkowska A., Kaczor J.J., Ziolkowski W., Flis D., Knap N., Kasperuk K., Antosiewicz J. (2018). hmSOD1 gene mutation-induced disturbance in iron metabolism is mediated by impairment of Akt signalling pathway. J. Cachex- Sarcopenia Muscle.

[9] Jacobs A., Worwood M. (1975). Ferritin in serum. Clinical and biochemical implications. N. Engl. J. Med..

[10] Kaczorowska-Hac B., Luszczyk M., Antosiewicz J., Ziolkowski W., Adamkiewicz-Drozynska E., Mysliwiec M., Milosz E., Kaczor J.J. (2017). HFE Gene Mutations and Iron Status in 100 Healthy Polish Children. J. Pediatr. Hematol..

[11] Kasprowicz K., Ziemann E., Ratkowski W., Laskowski R., Kaczor J.J., Dadci R., Antosiewicz J. (2013). Running a 100-km ultra-marathon induces an inflammatory response but does not raise the level of the plasma iron-regulatory protein hepcidin. J. Sports Med. Phys. Fit..

[12] Kautz L., Jung G., Valore E.V., Rivella S., Nemeth E., Ganz T. (2014). Identification of erythroferrone as an erythroid regulator of iron metabolism. Nat. Genet..

[13] Klausen T., Breum L., Fogh-Andersen N., Bennett P., Hippe E. (1993). The effect of short and long duration exercise on serum erythropoietin concentrations. Graefe’s Arch. Clin. Exp. Ophthalmol..

[14] Kortas J., Kuchta A., Prusik K., Prusik K., Ziemann E., Labudda S., Ćwiklińska A., Wieczorek E., Jankowski M., Antosiewicz J. (2017). Nordic walking training attenuation of oxidative stress in association with a drop in body iron stores in elderly women. Biogerontology.

[15] Antosiewicz J., Kortas J., Prusik K., Flis D., Leaver N., Ziemann E., Prusik K. (2015). Effect of Nordic Walking training on iron metabolism in elderly women. Clin. Interv. Aging.

[16] Lakka T.A., Nyyssönen K., Salonen J. (1994). Higher Levels of Conditioning Leisure Time Physical Activity are Associated with Reduced Levels of Stored iron in Finnish Men. Am. J. Epidemiol..

[17] Luo Y., Yoneda J., Ohmori H., Sasaki T., Kuniyasu H. (2014). Abstract 3380: Cancer usurps skeletal muscle as an energy repository. Mol. Cell. Biol..

[18] Nai A., Lidonnici M.R., Rausa M., Mandelli G., Pagani A., Silvestri L., Ferrari G., Camaschella C. (2015). The second transferrin receptor regulates red blood cell production in mice. Blood.

[19] Nai A., Pellegrino R.M., Rausa M., Pagani A., Boero M., Silvestri L., Saglio G., Roetto A., Camaschella C. (2014). The erythroid function of transferrin receptor 2 revealed by Tmprss6 inactivation in different models of transferrin receptor 2 knockout mice. Haematology.

[20] Nemeth E., Roetto A., Garozzo G., Ganz T., Camaschella C. (2005). Hepcidin is decreased in TFR2 hemochromatosis. Blood.

[21] Nemeth E., Tuttle M., Powelson J., Vaughn M.B., Donovan A., Ward D.M., Ganz T., Kaplan J. (2004). Hepcidin Regulates Cellular Iron Efflux by Binding to Ferroportin and Inducing Its Internalization. Science.

[22] Peeling P. (2010). Exercise as a mediator of hepcidin activity in athletes. Graefe’s Arch. Clin. Exp. Ophthalmol..

[23] Peeling P., Sim M., Badenhorst C., Dawson B., Govus A., Abbiss C., Swinkels R.W., Trinder D. (2014). Iron Status and the Acute Post-Exercise Hepcidin Response in Athletes. PLoS ONE.

[24] Roecker L., Meier-Buttermilch R., Brechtel L., Nemeth E., Ganz T. (2005). Iron-regulatory protein hepcidin is increased in female athletes after a marathon. Graefe’s Arch. Clin. Exp. Ophthalmol..

[25] Schwandt H.-J., Heyduck B., Gunga H.-C. (1991). Influence of prolonged physical exercise on the erythropoietin concentration in blood. Graefe’s Arch. Clin. Exp. Ophthalmol..

[26] Sullivan J.L. (2004). Is stored iron safe?. J. Lab. Clin. Med..

[27] Falzacappa M.V.V., Spasić M.V., Kessler R., Stolte J., Hentze M.W., Muckenthaler M.U. (2006). STAT3 mediates hepatic hepcidin expression and its inflammatory stimulation. Blood.

[28] Worwood M. (1998). Haemochromatosis. Int. J. Lab. Hematol..

[29] Zacharski L.R., Chow B.K., Howes P.S., Shamayeva G., Baron J.A., Dalman R.L., Malenka D.J., Ozaki C.K., Lavori P.W. (2008). Decreased Cancer Risk after Iron Reduction in Patients with Peripheral Arterial Disease: Results From a Randomized Trial. J. Natl. Cancer Inst..

[30] Zaloumis S., Allen K.J., Bertalli N.A., Turkovic L., Delatycki M.B., Nicoll A.J., McLaren C.E., English D.R., Hopper J., Giles G.G. (2015). Natural history of HFE simple heterozygosity for C282Y and H63D: A prospective 12-year study. J. Gastroenterol. Hepatol..

